# Risk-Aware Resource Management in Public Safety Networks

**DOI:** 10.3390/s19183853

**Published:** 2019-09-06

**Authors:** Panagiotis Vamvakas, Eirini Eleni Tsiropoulou, Symeon Papavassiliou

**Affiliations:** 1 School of Electrical and Computer Engineering, National Technical University of Athens, 15780 Athens, Greece (P.V.) (S.P.); 2 Department of Electrical and Computer Engineering, University of New Mexico, Albuquerque, NM 87131, USA

**Keywords:** resource management, unmanned aerial vehicles, risk, prospect theory, game theory, dynamic spectrum management, public safety networks

## Abstract

Modern Public Safety Networks (PSNs) are assisted by Unmanned Aerial Vehicles (UAVs) to provide a resilient communication paradigm during catastrophic events. In this context, we propose a distributed user-centric risk-aware resource management framework in UAV-assisted PSNs supported by both a static UAV and a mobile UAV. The mobile UAV is entitled to a larger portion of the available spectrum due to its capability and flexibility to re-position itself, and therefore establish better communication channel conditions to the users, compared to the static UAV. However, the potential over-exploitation of the mobile UAV-based communication by the users may lead to the mobile UAV’s failure to serve the users due to the increased levels of interference, consequently introducing risk in the user decisions. To capture this uncertainty, we follow the principles of Prospect Theory and design a user’s prospect-theoretic utility function that reflects user’s risk-aware behavior regarding its transmission power investment to the static and/or mobile UAV-based communication option. A non-cooperative game among the users is formulated, where each user determines its power investment strategy to the two available communication choices in order to maximize its expected prospect-theoretic utility. The existence and uniqueness of a Pure Nash Equilibrium (PNE) is proven and the convergence of the users’ strategies to it is shown. An iterative distributed and low-complexity algorithm is introduced to determine the PNE. The performance of the proposed user-centric risk-aware resource management framework in terms of users’ achievable data rate and spectrum utilization, is achieved via modeling and simulation. Furthermore, its superiority and benefits are demonstrated, by comparing its performance against other existing approaches with regards to UAV selection and spectrum utilization.

## 1. Introduction

Unmanned Aerial Vehicles (UAVs) have attracted great research and commercial interest due to their unique attributes to establish resilient and reliable communication during public safety threatening events [1], such as natural disasters and terrorist attacks. The UAVs are characterized by line-of-sight (LoS) communication links due to their ability to hover above a disaster-struck area, maneuver and adapt their altitude, as well as by several other additional degrees of freedom due to their controlled mobility [2]. The UAVs can be deployed in a flexible, fast, effortless and low-cost manner, while their usage can be dynamically adjusted, e.g., acting as receiver, caching node, mobile edge computing server, etc. [3]. Compared to other alternative strategies such as the ones that provide ground movable base stations [4], UAV-based solutions bypass associated deployment inefficiencies due to possible physical ground infrastructure damages, while allow for more efficient communication with the rest of system (i.e., backhauling).

The UAVs salient features to support the Public Safety Networks (PSNs) during a catastrophic event have already engaged many key industrial vendors to invest on their deployment. Facebook has launched the Aquila UAV to provide internet access to remote and non-accessible disaster-struck areas by the first-responders [5]. Google has invested on the Project Loon, where a UAV has provided emergency Long Term Evolution (LTE) coverage to Puerto Rico in the aftermath of the hurricane Maria [6]. Traditional PSN architectures that were based on dedicated cellular networks, such as the narrowband time-division multiple access (TDMA)-based Terrestrial Trunked Radio (TETRA) [7] and the Project 25 [8], require specialized hardware and offer low data rates [9], and consequently they are even less used nowadays [10]. On the other hand, in modern PSNs, UAVs are adopted to improve the wireless connectivity during a disaster, and this new reality motivates and demands the study of the dynamic spectrum management in UAV public safety networks, where the victims’ communication-related decisions are taken under risk and uncertainty, stemming primarily from the scarcity of the communication resources and the limited only information availability.

### 1.1. Related Work

Detailed research efforts have been devoted in the recent literature to the problem of resource management in UAV assisted PSNs targeting various objectives, such as network energy efficient operation, prolongation of mobile devices’ battery life [11], efficient bandwidth allocation to improve users’ achievable data rate, or even addressing simultaneously several of the aforementioned challenges [12,13].

In particular, in [14], the authors studies a UAV relay system, where the Non-Orthogonal Multiple Access (NOMA) technique facilitates the users’ communication with the UAV, and their goal is to maximize the total achievable throughput based on convex optimization while proposing a low-complexity heuristic algorithm. Following a similar philosophy, a centralized resource allocation approach is proposed in [15] towards maximizing the users’ total uplink minimum throughput in a specific time period of the UAV’s flight. The authors considered as additional constraints of the formulated optimization problem the system’s physical characteristics, such as the users’ devices’ energy availability and the UAV’s maximum speed.

Targeting at the users’ devices’ battery saving and the energy-efficient operation of the UAV-assisted PSN, the theory of minority games is used in [16] to form coalitions among the users residing in the PSN by exploiting their physical characteristics, e.g., distance from the UAV, energy availability, and determine their optimal transmission power to communicate with the UAV. Following the same pattern of creating users’ transmission coalitions to improve the energy efficiency of the PSN, a cluster formation mechanism is proposed in [17] based on the Chinese Restaurant Process and the users’ socio-physical characteristics. Given the users’ clusters, the authors introduced a non-cooperative power control problem to determine each user’s optimal transmission power in a distributed manner. Furthermore, in [18], the device-to-device communication is jointly exploited with the UAV networks to improve the users’ connectivity and the system’s energy-efficiency by targeting at optimally placing the mobile UAV. In [19], the problem of determining the optimal UAV’s position, the energy harvesting levels of the users from the UAV and each user’s optimal transmission power is studied via a game-theoretic approach, while a reinforcement learning mechanism is introduced to build users’ coalitions in the PSN.

More complex resource management problems in the UAV-assisted PSNs have also been examined in the recent literature. In [20], the authors formulated and solved a non-convex multi-variable optimization problem towards optimizing the users’ achievable data rate, their uplink transmission power, the network’s bandwidth usage, and the UAV’s position. In this examined problem, a UAV coexists with a Macro Base Station (MBS), while the UAV acts as a relay node to facilitate the communication of the users with the MBS. Moreover, the joint optimization problem of the users’ achievable uplink data rate and energy-efficiency is studied in [21], where the authors optimized the UAV system’s parameters related to its flight towards achieving the aforementioned goal. In [22], the authors determined the users’ optimal transmission power and time slots allocation in a UAV network via decomposing the joint total users’ rate optimization problem into the individual sub-problems of power allocation and transmission time slots allocation. The latter two optimization problems are solved sequentially and the one provides input to the other.

Although significant research efforts have been devoted to the resource management problem in UAV-assisted public safety networks, all the aforementioned research works implicitly or explicitly assume that the users in a PSN, i.e., victims and first responders, make decisions regarding their communication in a rational and risk-neutral utility-maximizing manner aiming at maximizing their perceived Quality of Service (QoS). However, in real-life, users are requested to make communication-related decisions based on uncertainty and risk, which, as mentioned above, stems from the resource-constrained communication environment and the partial only information availability. It should be clarified that, in this paper, the term user is utilized in a broader sense, practically representing an agent or algorithm located on the user’s mobile device, emulating a risk-aware behavior and making the corresponding communication decisions on behalf of a human. For the sake of simplicity of language and presentation, the term user is used throughout the paper to represent these entities when referring to making risk-aware decisions. Moreover, the current literature assumes that the users in a UAV-assisted PSN communicate exclusively either only with the UAV or the MBS, if the latter is still available in the disaster-struck area, without exploiting the potential of joint communication with both the UAV and the MBS, through their multi-communication interface devices. It is noted that advanced devices have already become available in the market in recent years [23,24], which can opportunistically access and utilize bandwidth resources even from distinct cells or providers. Such a multi-communication interface environment immensely modifies the flexibility enjoyed by the users who are not restricted in selecting only one receiver but can proportionally split their invested transmission power to multiple ones.

### 1.2. Contributions and Outline

**Figure 1:**
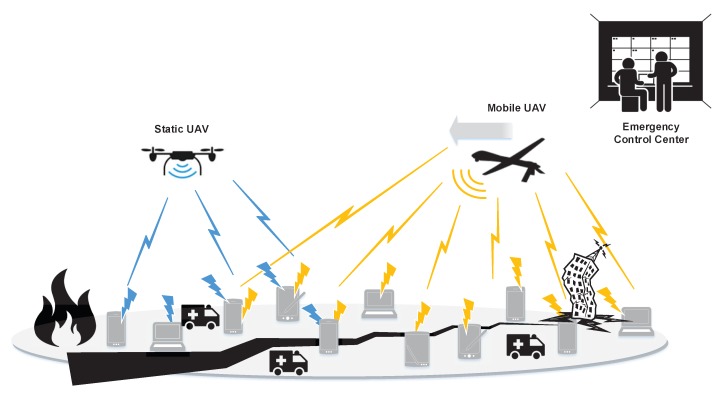
UAV-assisted public safety network topology.

Our paper aims at exactly filling these research gaps by proposing a holistic distributed approach to address the uplink energy-efficient resource management problem in UAV-assisted PSNs considering users’ risk-aware behavior in terms of communicating over a mobile UAV and/or a static UAV that hovers above the disaster-struck area. The static UAV hovers above an area in a specific altitude, i.e., 1000 m acting as a flying base station. Examples of static UAVs are the Boeing Insitu ScanEagle [25] and the Aerovel Flexrotor [26]. The mobile UAV also flies in a specific altitude, but closer to the end-users, i.e., 360 m. Examples of mobile UAVs are the AeroVironment RQ-11 Raven [27] and WASP AE Micro Air Vehicle [28]. The users exploit their devices’ dual communication interface to transmit their data to the two available receivers, i.e., the static and mobile UAVs, thus realizing a dynamic spectrum management. The static UAV hovers above the disaster area having a smaller portion of the total spectrum available to serve the users’ QoS requirements compared to the mobile UAV, based on the spectrum allocation that the Emergency Control Center (ECC) has planned. A representative high-level topology of the considered UAV-assisted PSN is presented in Figure 1. The aforementioned spectrum allocation is motivated by the fact that the mobile UAV has greater probability to fly closer to the users compared to the static UAV, thus the greater portion of allocated spectrum enables the mobile UAV to better serve the users, who have improved channel conditions communicating with the mobile UAV compared to the static UAV. In this setting, the mobile UAV is characterized as a Common Pool of Resources (CPR), where all the users are keen on opportunistically exploiting the communication with it enjoying the superior communication channel conditions and the greater portion of available bandwidth. However, if the users over-exploit their communication opportunities through the mobile UAV, then the mobile UAV will fail to serve the users’ QoS requests due to the increased interference observed. Specifically, if the users demonstrate risk-seeking behavior regarding their communication with the mobile UAV, they tend to increase their uplink transmission power levels to achieve a higher data rate and send more data through the mobile UAV. The latter phenomenon results in increased probability of failure of the mobile UAV due to the increased interference sensed at its receiver. Thus, at the point that the mobile UAV’s receiver cannot decode the users’ received signals, the probability of failure is considered equal to one, and the mobile UAV fails to serve the users. This consideration sets the physical limits of the mobile UAV’s failure. On the other hand, the static UAV is characterized as a safe resource, as the users receive a guaranteed QoS due to the low levels of interference in their communication with it and the static channel conditions, as the users are considered static in the disaster-struck area (Section 2).

The users demonstrate a risk-aware behavior towards deciding their power investment in their transmission to the static and/or the mobile UAV. Their risk-aware behavior stems from the uncertainty due to the finite available spectrum, and the probability of the mobile UAV failing to serve the opportunistic users due to the increased interference levels, as an outcome of the users’ over-exploiting their communication with the mobile UAV. The users’ risk-aware behavior in the considered uplink resource management and dynamic spectrum management problem is captured in appropriately designed prospect-theoretic utility functions following the paradigm of Prospect Theory (Section 3). A resource management framework is formulated as a maximization problem of each user’s expected prospect-theoretic utility function, and is addressed as a non-cooperative game among the users. The existence and uniqueness of a Pure Nash Equilibrium (PNE) regarding users’ power investment to the communication with the static and the mobile UAV is proven (Section 4). It is noted that the communication channel conditions among the users and the mobile UAV are superior compared to those with the static UAV. If the users demonstrate risk-averse behavior, they become more conservative in communicating with the mobile UAV (which is a shared communication resource among the users, i.e., Common Pool of Resources), thus they keep their uplink transmission power in low levels towards communicating with the mobile UAV [29]. Therefore, the interference introduced at the mobile UAV receiver is low, and the corresponding probability of failure of the mobile UAV is low. On the other hand, if the users demonstrate risk-seeking behavior, they tend to opportunistically and selfishly over-exploit their communication with the mobile UAV, resulting in high transmission power levels in their corresponding communication, which causes high levels of interference, and high values of the probability of failure, as the mobile UAV cannot decode their received signals due to the excessive interference.

An iterative distributed and low-complexity algorithm is introduced to determine the PNE, while its convergence to the PNE is shown (Section 5). A set of detailed simulations is presented to evaluate the performance of the proposed user-centric risk-aware resource management framework in UAV-assisted PSNs, in terms of achievable data rate, spectrum utilization, and superior performance compared to other approaches (Section 6). Finally, Section 7 concludes the paper.

## 2. System Model

A PSN is considered consisting of a static UAV *s* that hovers in a fixed position above the disaster-struck area, and a mobile UAV *m* that moves over the disaster area to better serve the users, i.e., victims and first responders. The disaster area has dimensions $L\times L[m^{2}]$ and the set of users is denoted as U={1,⋯,u,⋯,|U|}. Each user is assumed to be equipped with dual communication interface devices capable of transmitting data to the two available receivers (i.e., mobile and static UAV) simultaneously, if needed. The Emergency Control Center (ECC) allocates a portion *y* (where 0≤y≤1) of the overall available spectrum *W* to the static UAV, while the rest of the available spectrum for the public safety operations is allocated to the communication through the mobile UAV, i.e., (1−y)W. Typically a larger portion of spectrum is allocated to the mobile UAV compared to the static one, since, given the capability and flexibility of movement, the mobile UAV has better chances to be closer to the users compared to the static UAV. Noting that the mobile UAV flies at a lower altitude compared to the static UAV, the users are expected to experience superior channel conditions by communicating with the mobile UAV. The latter in combination with the greater available spectrum allocated to the mobile UAV, will result in improved QoS for the users that will be associated with the mobile UAV. Given this setting, the mobile UAV acts as a Common Pool of Resources (CPR) since all the users will be keen on communicating through it. Therefore, the mobile UAV may fail to serve users due to the increased levels of interference, if the users over-exploit their communication through the mobile UAV. On the other hand, the static UAV acts as a “safe resource” providing more predictable rewards to the users due to the lower levels of interference, as the users tend to invest low levels of transmission power to communicate with it due to the limited rewards (i.e., data rate) that they can achieve through this type of communication, and due to the fact that the channel conditions remain almost the same over the time given that both the UAV and the trapped users in the disaster area are static. A representative example is the Tham Luang Nang Non cave disaster event in Chiang Rai Province, Thailand, where a junior football team was trapped in a disaster area [30].

Each user *u* located in the disaster area has a distance $d_{u,i}\left[m\right]$, where i,i∈{s,m}, from the static UAV *s* and the mobile UAV *m*. The channel gain between the user *u* and the receiver *i* is defined as $G_{u,i}=\frac{k}{d_{u,i}^{2}}$, where *k* is a positive constant that expresses the channel fading for a time slot *t* (i.e., t=0.5 ms). The non-orthogonal multiple access (NOMA) technique is adopted for the communication with the static and the mobile UAV. The NOMA technique is considered as a major candidate for the upcoming 5G network deployments due to its high degree of bandwidth allocation flexibility and its compatibility with other access technologies and wireless systems. Under the NOMA technique, the users can access the network’s spectrum in each entirety instead of having a single and bandwidth-constrained resource block as in Orthogonal Multiple Access schemes, e.g., OFDMA. Thus, the users are able to dynamically exploit the bandwidth which is necessary for their transmission, hence offering superior spectral capacity since no part of the bandwidth remains idle [31]. Accordingly, the receivers perform the Successive Interference Cancellation (SIC) technique at the reception of the users’ signals. Without loss of generality and mainly for presentation purposes, the users’ channel gains are sorted as $G_{|U|,i}\leq \cdots \leq G_{u,i}\leq \cdots G_{1,i}$ and the corresponding sensed interference from the user *u* is denoted as $I_{u}=\left(P_{-u,i}\right)=\sum_{u^{\prime }\geq u+1}^{\left|U\right|}G_{u^{\prime },i}P_{u^{\prime }}+I_{0}$, where $I_{0}$ is the background noise and $P_{-u,i}$ is the transmission power vector of all the users communicating with the receiver *i* except for user *u*. It is noted that the users communicating with the static UAV do not sense the interference from the transmissions to the mobile UAV, as the static and the mobile UAV are considered to operate in different frequency bands of the spectrum, as explained above. Therefore, the user’s *u* signal-to-interference-plus-noise-ratio (SINR) as it is measured at the receiver i,i∈{s,m} is given as follows.

(1) $$\gamma_{u,i}\left(P_{u,i},P_{-u,i}\right)=\frac{G_{u,i}P_{u,i}}{I_{u}}$$

Given that each user *u* has a total uplink transmission power $P_{u}^{Max}$, its goal is to determine the power investment portion (i.e., distribution) that will be allocated for the communication with the static and the mobile UAV. Letting $x_{u},x_{u}\in \left[0,1\right]$ denote the percentage of user’s power investment to the communication with the mobile UAV, then its corresponding transmission power is $x_{u}P_{u}^{Max}$, and consequently the corresponding transmission power for the communication with the static UAV is $\left(1-x_{u}\right)P_{u}^{Max}$. At this point, it should be clarified that the problem of optimally positioning the mobile UAV is not part of our study, as much work has already been devoted to the optimal UAV positioning [2,3]. At each time slot *t*, the mobile UAV changes position, and its coordinates are considered to be known in the following analysis.

## 3. Capturing User’s Behavior via Prospect Theory

The problem of users’/victims’ risk-aware decision making during disaster events has been extensively studied in the literature of Public Safety Systems. Current research on Public Safety Systems dealing with dynamically evolving disaster scenarios focuses mostly on the crowd evacuation dynamics. While macroscopic models treat the crowd as a flowing continuum based on the physical features of liquid flow [32], microscopic models based on the cellular automata model [33] and social force model [34], treat the human as a self-driving particle. Indeed, human behavioral models capture individuals’ characteristics and peculiarities during disasters. For example, the Nomad model focuses on humans’ daily activities [35]; the social identity theory recognizes the impact of belonging to a group on human behavior [36]; and the elaborated social identity model identifies that human social behavior can rapidly change during an emergency [37]. Self-organization phenomena have also been studied in the context of Public Safety Systems [38], such as the herding effect [39], zipper effect [40], Faster-is-Slower effect [41], and so on. In this paper, we focus on the users’/victims’ risk-aware decisions regarding their communication with the static and/or the mobile UAV by adopting the principles of Prospect Theory. The proposed framework is in line with the existing research initiative of 5G networks to support users’ autonomy and develop user-centric approaches that will be implemented in a distributed manner without requiring a centralized entity to control the communication environment and impose additional signaling overhead [42].

The considered wireless communication environment is characterized by high levels of uncertainty, stemming from the dynamically changing communication, the probability of the mobile UAV to be unable to serve the users due to its over-exploitation, i.e., increased levels of interference, and the partial available information to the users. Under this uncertain environment, the users are making decisions under risk regarding their communication with the UAVs based on their behavioral characteristics.

Towards capturing the users’ risk-aware behavior, we follow the principles of Prospect Theory. Prospect Theory was introduced by Kahneman and Tversky in 1979 [43], where humans make autonomous risk-aware decisions under uncertainty, which are associated with the unpredictable payoff of their choices that is evaluated with some probability. Following the concept of Prospect Theory, the users experience greater dissatisfaction from a potential outcome of losses, compared to their corresponding satisfaction from gains of the same amount. In addition, the users’ prospect-theoretic payoff, i.e., utility, is estimated with respect to a reference point $U_{0,u}$, which acts as the ground truth for users’ satisfaction. Based on the above, the user’s prospect-theoretic utility can be defined as follows

(2) $$U_{u}\left(x_{u},x_{T}\right)=\left\{\begin{array}{cc} {\left(U_{u}-U_{0,u}\right)}^{a_{u}}, & if\;U_{u}>U_{0,u}\\ -k_{u}{\left(U_{0,u}-U_{u}\right)}^{b_{u}}, & otherwise \end{array}\right.\;$$

where $U_{u}\left(x_{u},x_{T}\right)$ is the user’s *u* actual utility expressed as the user’s achieved energy efficiency and $x_{T}=\sum_{u=1}^{\left|U\right|}x_{u}$ is the total power investment of all the users to their communication with the mobile UAV. The reference point $U_{0,u}$ is defined as the user’s *u* achieved energy efficiency if the user was only exploiting its communication with the static UAV and is given as follows.

(3) $$U_{0,u}=\frac{y\cdot W\cdot log(1+\gamma_{u,s}\left(P_{u,s},P_{-u,s}\right))}{P_{u,s}}$$

The user *u* has the potential to experience superior actual perceived utility $U_{u}$ by opportunistically exploiting its communication and data transmission to the mobile UAV. In this case, the user achieves greater payoff than the reference point and thus, the user enjoys improved performance and in a personalized manner (through parameters $a_{u},b_{u}$, and $k_{u}$ explained below), as expressed by the first branch of Equation (2). However, if the users cumulatively over-exploit their communication to the mobile UAV by investing increased transmission power, then the levels of interference at the receiver increases, thus the user can experience less payoff than the reference point, i.e., loss, as expressed by the second branch of Equation (2).

The way that each user experiences its personalized gains and losses, as well as how much risk-seeking it can become regarding its transmission power investment for the data transmission to the mobile UAV, are captured by the user’s personalized behavioral parameters $a_{u},b_{u}$, and $k_{u}$. In particular, the parameters $a_{u}$ and $b_{u}$, $a_{u},b_{u}\in \left(0,1\right]$ capture the users’ perception of the gains and losses, respectively. A user becomes more risk-seeking and tends to invest more transmission power to the mobile UAV communication for increasing values of $a_{u}$. In addition, for decreasing values of $b_{u}$, the user experiences greater dissatisfaction from its losses, thus it becomes more risk-averse. Without loss of generality, in the following, we assume $a_{u}=b_{u}$. The loss aversion parameter $k_{u},k_{u}\in \left[0,+\infty \right)$ reflects the impact of losses compared to gains on user’s prospect-theoretic utility. If $k_{u}>1$, the user *u* weighs the losses more than the gains, while, if $0\leq k_{u}\leq 1$, the user weighs more or equal the gains than the losses, thus presenting an aggressive gain seeking behavior.

As mentioned above, each user exploits its dual communication interface in order to transmit its data to the static and the mobile UAV via intelligently investing its transmission power, and dynamically managing the available spectrum. Therefore, the user’s actual utility is given as follows.

(4) $$U_{u}\left(x_{u},x_{T}\right)=U_{0,u}\left(1-x_{u}\right)+F_{u}\cdot x_{u}\cdot RoR\left(x_{T}\right)$$

The first term of Equation (4) expresses the user’s utility, in terms of energy-efficiency units, by its transmission to the static UAV. The second term of Equation (4) captures the user’s satisfaction by transmitting its data to the mobile UAV, where $F_{u}=\frac{\left(1-y\right)\cdot W\cdot log(1+\gamma_{u,m}\left(P_{u,m},P_{-u,m}\right))}{P_{u,m}}$. The function $RoR(x_{T})$ expresses the Rate of Return (RoR) of the mobile UAV-based communication and is a decreasing function with respect to the users’ total power investment for communication with the mobile UAV. The latter is justified and motivated by the fact that, as users’ increase their transmission power investment to their mobile UAV-based communication, the mobile UAV is less able to serve them due to the increased interference at the reception of their signals and its limited spectrum. Without loss of generality, we adopt a representative rate of return function.

(5) $$RoR\left(x_{T}\right)=2-e^{x_{T}-1}$$

Following the previous reasoning, the mobile UAV has a probability of not being able to serve the users (referred to as probability of failure), which is an increasing function with respect to the users’ total transmission power investment to their mobile UAV-based communication. For demonstration purposes and without limiting the following analysis, we adopt a probability of failure function $P(x_{T})$ for the mobile UAV, as follows: $P\left(x_{T}\right)=x_{T}^{2}$. If the mobile UAV is able to serve the users, then each user perceives an actual utility $U_{u}$ greater than the reference point, i.e., the user gains from investing to the mobile UAV-based communication. Thus, by calculating the difference $U_{u}-U_{0,u}$ via Equations (3) and (4), we have $U\left(x_{u},x_{T}\right)=x_{u}^{a_{u}}\cdot {\left[F_{u}\cdot RoR\left(x_{T}\right)-U_{0,u}\right]}^{a_{u}}$. For simplicity of notation, we normalize the rate of return function, so that $U_{0,u}=1$, and denote $\bar{RoR_{u}}\left(x_{T}\right)≜{\left(F_{u}\cdot RoR\left(x_{T}\right)-1\right)}^{a_{u}}$, where $\bar{RoR_{u}}\left(x_{T}\right)$ is assumed concave, decreasing, twice continuously differentiable and positive. Thus, we conclude that $U_{u}\left(x_{u},x_{T}\right)=x_{u}^{a_{u}}\bar{RoR_{u}}\left(x_{T}\right)$. In the opposite case that the mobile UAV is not able to serve the users, the user achieves less utility than its reference point. Thus, by calculating the difference ($U_{0,u}-U_{u}$), and shaping the result following the second branch of Equation (2), we have $U_{u}\left(x_{u},x_{T}\right)=-k_{u}x_{u}^{a_{u}}$. In this case, the second term of Equation (4) is zero, as the user gets zero satisfaction from the mobile UAV-based communication, even if invested some power in this communication effort.

Summarizing the above analysis, the users’ prospect-theoretic utility function is written as follows.

(6) $$U_{u}\left(x_{u},x_{T}\right)=\left\{\begin{array}{cc} x_{u}^{a_{u}}\bar{RoR_{u}}\left(x_{T}\right), & if\;U_{u}>U_{0,u}\\ -k_{u}x_{u}^{a_{u}}, & otherwise \end{array}\right.$$

Furthermore, by incorporating the probability of the mobile UAV to fail serving the users’ QoS requests, the user’s prospect-theoretic utility is reshaped as follows.

(7) $$U_{u}\left(x_{u},x_{T}\right)=\left\{\begin{array}{cc} x_{u}^{a_{u}}\bar{RoR_{u}}\left(x_{T}\right), & with\;probability\;(1-P\left(x_{T}\right))\\ -k_{u}x_{u}^{a_{u}}, & with\;probability\;P(x_{T}) \end{array}\right.$$

## 4. Risk-Aware Resource Management in UAV Networks

### 4.1. Problem Formulation

In this section, we formulate the distributed resource management problem to enable the users, to autonomously determine their uplink transmission power investment to the static and mobile UAV-based communication. This in turn allows the realization of a dynamic spectrum management. Given the probabilistically defined user’s prospect-theoretic utility function (Equation (7)), the user’s expected prospect-theoretic utility function is derived as follows.

(8) $$E\left(U_{u}\right)=x_{u}^{a_{u}}\bar{RoR_{u}}\left(x_{T}\right)\left(1-P\left(x_{T}\right)\right)-k_{u}x_{u}^{a_{u}}P\left(x_{T}\right)$$

Therefore, the corresponding distributed resource management problem is formulated as a maximization problem of each user’s expected prospect-theoretic utility function, as follows.

(9) $$\begin{array}{c} \max_{x_{u}\in \left[0,1\right]}\left\{E\left(U_{u}\right)=x_{u}^{a_{u}}h_{u}\left(x_{T}\right)\right\},\;\forall u\in U \end{array}$$

where $h_{u}\left(x_{T}\right)=\bar{RoR_{u}}\left(x_{T}\right)\left(1-P\left(x_{T}\right)\right)-k_{u}P\left(x_{T}\right)$ and $h_{u}\left(x_{T}\right)$ is the effective rate of return of the mobile UAV-based communication considering the users’ personal behavioral characteristics and the probability of the mobile UAV to fail serving the users.

The maximization problem of Equation (9) can be addressed as a non-cooperative game among the users. The non-cooperative game is defined as $G=[U,{\left\{X_{u}\right\}}_{u\in U},{\left\{E\left(U_{u}\right)\right\}}_{u\in U}]$, where *U* denotes the set of users, $X_{u}=\left[0,1\right],\forall u\in U$ is the strategy space of user *u* (i.e., its percentage power investment to the mobile UAV-based communication), and $E(U_{u})$ is the user’s payoff as expressed by its expected prospect-theoretic utility function (Equation (8)). Our goal is to determine the existence and uniqueness of a Pure Nash Equilibrium (PNE) point representing users’ power investment to their mobile UAV-based communication. The PNE is denoted as $x^{*}=\left[x_{1}^{*},\cdots ,x_{u}^{*},\cdots ,x_{\left|U\right|}^{*}\right]$ and at the PNE point no user can further improve its achieved expected prospect-theoretic utility by unilaterally changing its uplink transmission power investment given the strategies of the rest of the users, i.e., $E\left(U_{u}\left(x_{u}^{*},x_{-u}^{*}\right)\right)\geq E\left(U_{u}\left(x_{u},x_{-u}^{*}\right)\right),\;\forall x_{u}\in X_{u}$. Before proving the existence and uniqueness of the PNE point in the following subsection, we provide some useful mathematical properties regarding the probability of failure $P(x_{T})$ and the normalized rate of return function $\bar{RoR_{u}}\left(x_{T}\right)$. The probability of the mobile UAV failing to serve the users is strictly increasing, twice differentiable and convex in the normalized total power investment $x_{T}=\left[0,1\right]$ and $P(x_{T}\geq 1)=1$. The normalized rate of return function $\bar{RoR_{u}}\left(x_{T}\right)≜{\left(F_{u}\cdot RoR\left(x_{T}\right)-1\right)}^{a_{u}}$ is twice-differentiable, monotonic decreasing ($\frac{\partial \bar{RoR_{u}}\left(x_{T}\right)}{\partial x_{T}}<0$), concave ($\frac{\partial^{2}\bar{RoR_{u}}\left(x_{T}\right)}{\partial x_{T}^{2}}<0$), and positive $\forall x_{T}\in \left[0,1\right]$.

### 4.2. Problem Solution

In this section, we prove the existence and uniqueness of the PNE for the game *G*, as well as the convergence of all the users’ strategies to the PNE. The concept of best response strategy $BR_{u}\left(x_{-u}\right)$ is adopted, where $BR_{u}\left(x_{-u}\right)=argmaxE\left(U_{u}\right),BR_{u}:\bar{X_{-u}}⇉X_{u}$, where $\bar{X_{-u}}$ denotes the aggregate investment of all the users except for user *u*. The users’ best response strategy $BR_{u}$ lies in the interval [0,1], where $BR_{u}\left(x_{-u}\right)=0$ means that the user communicated only through the static UAV and, if $BR_{u}\left(x_{-u}\right)=1$, then the user invested its maximum uplink transmission power to the communication with the mobile UAV. In the following theorem, we examine the properties of the effective rate of return function $h_{u}\left(x_{T}\right)=\bar{RoR_{u}}\left(x_{T}\right)\left(1-P\left(x_{T}\right)\right)-k_{u}P\left(x_{T}\right)$.

**Theorem** **1.**  *The effective rate of return function $h_{u}\left(x_{T}\right)$ is decreasing, concave and positive in the modified strategy space $X_{u}^{\prime }=\left[0,\mu \right],\;\forall a_{u}<0.5$.*

**Proof.**  The first-order derivative of $h_{u}\left(x_{T}\right)$ with respect to each user’s power investment $x_{u}$ is given as follows.

(10) $$\frac{\partial h_{u}\left(x_{T}\right)}{\partial x_{u}}=\frac{\partial \bar{RoR_{u}}\left(x_{T}\right)}{\partial x_{u}}\left(1-x_{T}^{2}\right)-2x_{T}\bar{RoR_{u}}\left(x_{T}\right)-2k_{u}x_{T}$$

Based on Equation (5) and the normalized rate of return function $\bar{RoR_{u}}\left(x_{T}\right)={\left(F_{u}\cdot RoR\left(x_{T}\right)-1\right)}^{a_{u}}$, we have $\bar{RoR_{u}}\left(x_{T}\right)>0$ and $\frac{\partial \bar{RoR_{u}}\left(x_{T}\right)}{\partial x_{T}}<0$. Thus, $\frac{\partial h_{u}\left(x_{T}\right)}{\partial x_{u}}<0$ and the effective rate of return function is decreasing. Considering the second-order derivative of $h_{u}\left(x_{T}\right)$, we have:

(11) $$\frac{\partial^{2}h_{u}\left(x_{T}\right)}{\partial x_{u}^{2}}=\frac{\partial^{2}\bar{RoR_{u}}\left(x_{T}\right)}{\partial x_{u}^{2}}\left(1-x_{T}^{2}\right)+g\left(x_{T}\right)-2k_{u}$$

where $g\left(x_{T}\right)=-4x_{T}\frac{\partial \bar{RoR_{u}}\left(x_{T}\right)}{\partial x_{u}}-2\bar{RoR_{u}}\left(x_{T}\right)$. Given that $\frac{\partial^{2}\bar{RoR_{u}}\left(x_{T}\right)}{\partial x_{T}^{2}}<0$, the normalized aggregate investment is $x_{T}\leq 1$, and $g(x_{T})<0$ in $X_{u},\;\forall a_{u}<0.5$, thus we show that $\frac{\partial^{2}h_{u}\left(x_{T}\right)}{\partial x_{u}^{2}}<0$. Therefore, the effective rate of return function $h_{u}\left(x_{T}\right)$ is concave.

Towards showing that the effective rate of return function $h_{u}\left(x_{T}\right)$ is positive, we apply Bolzano’s Theorem within $X_{u}=\left[0,1\right]$ which is an important specialization of Intermediate Value Theorem [44]. We observe that $h_{u}\left(0\right)>0$ and $h_{u}\left(1\right)<0$, hence there exists a value $\mu \in X_{u}$, such that $h_{u}\left(\mu \right)=0$. Thus, $h_{u}$ is positive in the modified strategy space $X_{u}^{\prime }=\left[0,\mu \right]\subseteq \left[0,1\right]=X_{u}$. □

In the following theorem, we prove the existence of the PNE for the non-cooperative game *G*.

**Theorem 2.** ***(Existence of PNE)***  For the non-cooperative game $G=[U,{\left\{X_{u}\right\}}_{u\in U},{\left\{E\left(U_{u}\right)\right\}}_{u\in U}]$, there exists a PNE $x_{u}^{*},x_{u}^{*}\in X_{u}^{\prime },\forall u\in U$.

**Proof.**  Initially, we examine the first-order derivative of the user’s expected prospect-theoretic utility $E(U_{u}\left(x_{u},x_{T}\right))$, as follows.

(12) $$\frac{\partial E(U_{u}\left(x_{u},x_{T}\right))}{\partial x_{u}}={x_{u}}^{a_{u}-1}\left(x_{u}\frac{\partial h_{u}\left(x_{T}\right)}{\partial x_{T}}+a_{u}h_{u}\left(x_{T}\right)\right)$$

For $x_{u}=0$, we have $\left(x_{u}\frac{\partial h_{u}\left(x_{T}\right)}{\partial x_{T}}+a_{u}h_{u}\left(x_{T}\right)\right){|}_{x_{u}=0}>0$, since $h_{u}\left(x_{T}\right)>0$. Considering a very small value $\epsilon_{1}\to 0,\epsilon_{1}>0$, we have $\left(x_{u}\frac{\partial h_{u}\left(x_{T}\right)}{\partial x_{T}}+a_{u}h_{u}\left(x_{T}\right)\right){|}_{x_{u}=\epsilon_{1}}>0$, thus $\frac{\partial E(U_{u}\left(x_{u},x_{T}\right))}{\partial x_{u}}{|}_{x_{u}=\epsilon_{1}}>0$. For a very large value $\epsilon_{2}=\mu \in X_{u}^{\prime }$, we have $h_{u}\left(\epsilon_{2}\right)=0$, thus $\left(x_{u}\frac{\partial h_{u}\left(x_{T}\right)}{\partial x_{T}}+a_{u}h_{u}\left(x_{T}\right)\right){|}_{x_{u}=\epsilon_{2}}<0$, and subsequently $\frac{\partial E(U_{u}\left(x_{u},x_{T}\right))}{\partial x_{u}}{|}_{x_{u}=\epsilon_{2}}<0$. Given that $\frac{\partial E(U_{u}\left(x_{u},x_{T}\right))}{\partial x_{u}}{|}_{x_{u}=\epsilon_{1}}>0$ and $\frac{\partial E(U_{u}\left(x_{u},x_{T}\right))}{\partial x_{u}}{|}_{x_{u}=\epsilon_{2}}<0$, and by applying the Intermediate Value Theorem, we prove that there exists at least one $x_{u}^{*}$ value, $x_{u}^{*}\in X_{u}^{\prime }$, such that $\frac{\partial E(U_{u}\left(x_{u},x_{T}\right))}{\partial x_{u}}{|}_{x_{u}=x_{u}^{*}}=0$. Given also the properties of $P(x_{T})$ and $\bar{RoR_{u}}\left(x_{T}\right)$, as discussed in Section 4.1, we conclude that $x_{u}^{*},x_{u}^{*}\in X_{u}^{\prime },\forall u\in U$ is a PNE of the game $G=[U,{\left\{X_{u}\right\}}_{u\in U},{\left\{E\left(U_{u}\right)\right\}}_{u\in U}]$. □

In the following theorem, we prove the uniqueness of the PNE point for the game *G*.

**Theorem 3.** ***(Uniqueness of PNE)***  The PNE point $x_{u}^{*},x_{u}^{*}\in X_{u}^{\prime },\forall u\in U$ of the non-cooperative game $G=[U,{\left\{X_{u}\right\}}_{u\in U},{\left\{E\left(U_{u}\right)\right\}}_{u\in U}]$ is unique.

**Proof.**  Initially, we study the concavity of $E(U_{u}\left(x_{u},x_{T}\right))$ by examining its second-order derivative with respect to $x_{u}$.

(13) $$\frac{\partial^{2}E\left(U_{u}\left(x_{u},x_{T}\right)\right)}{\partial x_{u}^{2}}=a_{u}\left(a_{u}-1\right){x_{u}}^{a_{u}-2}h_{u}\left(x_{T}\right)+2a_{u}{x_{u}}^{a_{u}-1}\frac{\partial h_{u}\left(x_{T}\right)}{\partial x_{u}}+{x_{u}}^{a_{u}}\frac{\partial^{2}h_{u}\left(x_{T}\right)}{\partial x_{u}^{2}}$$

All the terms of the above equation are negative given that $x_{u}>0\in X_{u}^{\prime }$, $h_{u}$ is positive, decreasing, and concave in $X_{u}^{\prime }$, and $a_{u}<0.5$. Thus, we conclude that $\frac{\partial^{2}E\left(U_{u}\left(x_{u},x_{T}\right)\right)}{\partial x_{u}^{2}}<0$, therefore $E(U_{u}\left(x_{u},x_{T}\right))$ is concave. Based on the above analysis, the point $x_{u}^{*},x_{u}^{*}\in X_{u}^{\prime },\forall u\in U$ is a unique global maximum of $E(U_{u}\left(x_{u},x_{T}\right))$ and a unique PNE point of the non-cooperative game $G=[U,{\left\{X_{u}\right\}}_{u\in U},{\left\{E\left(U_{u}\right)\right\}}_{u\in U}]$. □

Towards providing the convergence of the users’ strategies of uplink transmission power to the PNE point, we use the best response dynamics $BR_{u}\left(x_{-u}\right)$. In the following theorem, we prove that the users’ best response dynamics $BR_{u}\left(x_{-u}\right),\forall u\in U$ monotonically decrease with users’ aggregate power investment $x_{T}$ and converge to the PNE point.

**Theorem 4.** ***(Convergence to PNE)***  The user’s best response strategy $BR_{u}\left(x_{-u}\right),\forall u\in U$ in the non-cooperative game $G=[U,{\left\{X_{u}\right\}}_{u\in U},{\left\{E\left(U_{u}\right)\right\}}_{u\in U}]$ is decreasing in $x_{T}$ and converges to the PNE point $x_{u}^{*},x_{u}^{*}\in X_{u}^{\prime },\forall u\in U$.

**Proof.**  Let $H\left(x_{T}\right)=-a_{u}\frac{h_{u}\left(x_{T}\right)}{MM\partial h_{u}\left(x_{T}\right)\;/\;\partial x_{u}}$ be defined as the optimal non zero investment of each user u,u∈U, where $H\left(BR_{u}\left(x_{-u}\right)+x_{-u}\right)=BR_{u}\left(x_{-u}\right),$ when $BR_{u}\left(x_{-u}\right)>0$. It is easily shown that $\frac{\partial H(x_{T})}{\partial x_{u}}<0$, thus, *H* is monotonically decreasing in $x_{u}$. Let now $x_{1}=BR_{u}\left(x_{-1}\right)$, $x_{2}=BR_{u}\left(x_{-2}\right)$, with $x_{-1},x_{-2}\in {\bar{X^{\prime }}}_{-u}$. If $BR_{u}$ is increasing, then for $x_{2}>x_{1}$, then $BR_{u}\left(x_{-2}\right)>BR_{u}\left(x_{-1}\right)$. However, since *H* is decreasing, for $x_{2}>x_{1}$, $H\left(BR_{u}\left(x_{-2}\right)+x_{-2}\right)=BR_{u}\left(x_{-2}\right)<BR_{u}\left(x_{-1}\right)=H\left(BR_{u}\left(x_{-1}\right)+x_{-1}\right)$, which is contradicting. Subsequently, we conclude that best response $BR_{u}$ is decreasing in $x_{T}$, and the users’ strategies converge to the game’s *G* unique PNE. □

## 5. Distributed Algorithm—DYNAMISM

In the following, we present and discuss a distributed and low complexity algorithm, namely **DYNAMI**c **S**pectrum **M**anagement in risk-aware UAV networks (i.e., DYNAMISM), which undertakes the practical implementation of the previously described theoretical framework. DYNAMISM algorithm acts as a common interface which enables the optimal user’s power investment determination for the spectrum usage of both the UAVs. Transmission is differentiated for each user given its relative position from both UAVs, with the static UAV to provide a stable channel gain environment due to its steady position, while the mobile UAV offers varying channel gain conditions since its movement impacts the quality of communication among itself and the users. DYNAMISM algorithm is executed per timeslot, with its duration range (i.e., 0.5 msec) to allow capturing a snapshot of the network’s operation. Hence, the users are able to optimize their power investment to each UAV’s spectrum during this timeslot, an outcome which may be modified in the upcoming timeslots, if the transmission via the mobile UAV becomes more favorable or strenuous.

The algorithm promotes a highly decentralized approach with regards to decision making, since each user is responsible for streamlining its power investment in order to optimize its prospect-theoretic expected utility during the resource allocation process. At the beginning of the algorithm’s implementation, the users define their behavioral characteristics reflecting their QoS preferences and their perceptions towards risk. Based on their topological and prospect-theoretic modeling, the users identify the optimal power investment to each UAV’s spectrum, starting from any initial feasible point. Eventually, the algorithm converges into an optimal allocation of users’ power investment among the UAVs indicating a successful transmission, otherwise if excessive congestion and over-exploitation of mobile UAV’s spectrum is identified, then the algorithm will terminate and only the users who transmitted via the static UAV will be able to communicate. During the entire process, the role of the system administrator is rather limited, as only the overall interference in the network is required to be exchanged from the UAVs to the users. The user-centric design of the DYNAMISM algorithm and the parallel execution of actions significantly mitigate the computational complexity required to determine the game’s optimal operational point (i.e., Pure Nash Equilibrium). The basic steps and actions of DYNAMISM algorithm are summarized in Algorithm 1.

**Algorithm 1** DYNAMISM: DYNAMIc Spectrum Management in risk-aware UAV networks.**Require:**   constants $k_{u}$, $a_{u}$, ϵ; user and UAV position coordinates; UAV spectra $W_{UAV}^{mobile}=\left(1-y\right)W$, $W_{UAV}^{static}=yW$ 1:ite←1; $convergence^{\left(ite\right)}\leftarrow 0$; systemfail←0  2:Calculate Channel Gains per user and apply SIC  3:Assign initial random $x_{u}^{\left(ite\right)}$  4:**while**$convergence^{\left(ite\right)}$ = 0 **do**5:  Calculate $P_{u,s},P_{u,m}$;  6:  Overall interference per UAV broadcasted and each user calculates its own sensed interference  7:  Calculate utility $E{\left(U_{u}\right)}^{\left(ite\right)}$  8:  **for all** $x_{u}\in \left[0,1\right]$ **do**
9:    $x_{u}^{*}$ = $argmax_{x_{u}}$$E(U_{u})$  10:    **if** $E\left(U_{u}\right)>E{\left(U_{u}\right)}^{\left(ite\right)}$ **then**
11:      $x_{u}^{\left(ite+1\right)}$←$x_{u}^{*}$ **and** $E{\left(U_{u}\right)}^{\left(ite+1\right)}$←$E(U_{u})$  12:    **end if** 13:  **end for** 14:  Calculate $mobile\;UAV\;Spectrum\;utilization\;util=\frac{\sum_{u=1}^{\left|U\right|}\left(1-y\right)\cdot W\cdot log\left(1+\gamma_{u,m}\left(P_{u,m},P_{-u,m}\right)\right)}{(1-y)W}$  15:  **if** util>1 **then**
16:    systemfail←1  17:  **end if** 18:  **if** $x_{u}^{\left(ite+1\right)}-x_{u}^{\left(ite\right)}<\epsilon$ **then**
19:    $convergence^{\left(ite+1\right)}\leftarrow 1$  20:  **end if** 21:  ite←ite+1  22:**end while** 23:**return**The DYNAMISM algorithm returns each user’s investment $x_{u}$, if the mobile UAV achieves to serve the users and the flag “systemfail”, if the mobile UAV fails to serve the users.

The low duration of each timeslot ensures that the movement of the mobile UAV within the timeslot duration is rather limited with no significant impact on the channel gain determination. However, since DYNAMISM algorithm is timeslot based, each iteration for a consecutive timeslot is capable of capturing the trajectory of the mobile UAV, and repeat the resource allocation process for the new topological coordinates. Thus, the algorithm manages to address the challenge of changing positions of the UAV above the users and identify the optimal power investment and spectrum allocation for the system.

## 6. Performance Evaluation

In this section, we present a detailed evaluation of the performance and the operational characteristics of the proposed framework, obtained via modeling and simulation. Furthermore, a detailed comparative evaluation of the proposed framework against other approaches with respect to user selection policies to multiple UAV spectrum sources, is provided. All simulations were conducted under the MATLAB computing environment on an Intel(R) Core(TM) i7-7500U CPU @ 2.70 GHz 2.90 GHz laptop with 8.00 GB RAM.

In the tested operational scenario, we assumed a wireless network covering an area of radius ℜ=3.5 km, and consisting of two UAV aircrafts flying above a number of |U|=20 ground users randomly distributed within the wireless network. The first UAV (i.e., static UAV) is hovering in a steady position and provides stable transmission conditions to the users of the network, while the second UAV (i.e., mobile UAV) moves above the users resulting in changing channel gains depending on its relative position towards them. For demonstration purposes, we assumed that the spectrum of the network is W=4 MHz, 80% of which is allocated to the mobile UAV and 20% allocated to the static UAV. Both UAVs operate under the NOMA transmission paradigm, and acknowledging system’s physical limitations, user maximum feasible transmission power is set at $P_{u}^{Max}=0.2$ Watts, being split between the communication with the two UAVs.

We examined the system’s behavior under various transmission scenarios considering initially homogeneous population (Section 6.1.1) where all users present common behavior characteristics; subsequently, (Section 6.1.2) a heterogeneous set of users was considered where the impact of diversifying risk characteristics—as modeled via Prospect Theory—was evaluated. Moreover the impact of the mobility and repositioning of the mobile UAV on the achievable user data rate and system spectrum utilization was evaluated (Section 6.1.3). Finally, we illustrate some comparative results in order to assess the performance of the DYNAMISM algorithm against two other alternative approaches: one performing a fixed user allocation to each UAV under the Expected Utility Theory without considering risk behavioral modeling, and a second one providing exclusive UAV selection by enabling the users’ devices to select each UAV based on their most favorable channel conditions at each timeslot (Section 6.2). The key simulation parameters that have been adopted in the following numerical results are summarized in Table 1.

### 6.1. Risk-Aware Dynamic Spectrum Management

#### 6.1.1. Homogeneous Population: Common User Behavior

**Figure 2:**
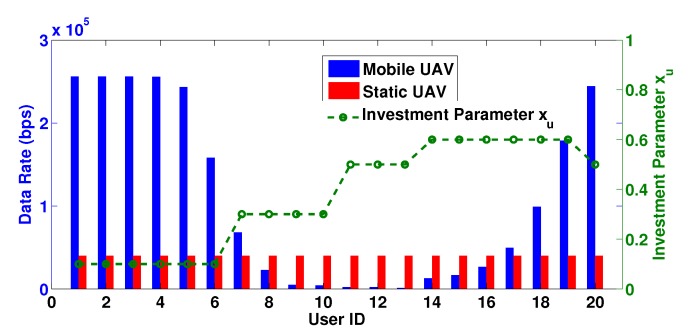
Data rates and power investment per user ID.

Initially, we considered that the users have common prospect-theoretic parameters, i.e. the same risk aversion parameter $k_{u}=20$ and sensitivity parameter $a_{u}=0.1$. Figure 2 depicts users’ achievable data rates from each UAV, as well as the relative investment $x_{u}$ to the mobile UAV, for a case where both UAVs are placed very close to each other. Please also note that, for presentation purposes, the user IDs are assigned such that increasing user ID corresponds to an increasing distance from the mobile UAV. We observed that, due to the higher spectrum availability from the mobile UAV, the obtained data rates for users who are very close to the mobile UAV or far away from it are quite high, owing either to their very favorable channel gain conditions (for the close ones) or to the absence of interference as a consequence of the application of SIC technique (for the distant ones) adopted by NOMA. On the contrary, middle distance users are severely impacted by their worsening channel conditions in conjunction with the rising interference levels sensed from the users with worse channel gains than them. The overall above discussion is also reflected on the values of the power investment parameter, with the users close to the mobile UAV to obtain high data rates even with small investment values. Distant users on the contrary do not further increase their investment significantly (e.g., observe for example users with IDs 14–20), since they manage to achieve satisfactory communication with the mobile UAV due to the low sensed interference as explained above. The static UAV, since it operates under a stricter framework with each user transmitting with stable channel conditions, delivers much more stable achievable data rate to all users, with all of them managing to split the limited spectrum in a more balanced manner.

#### 6.1.2. Heterogeneous Population: Diversifying User Behavior

**Figure 3:**
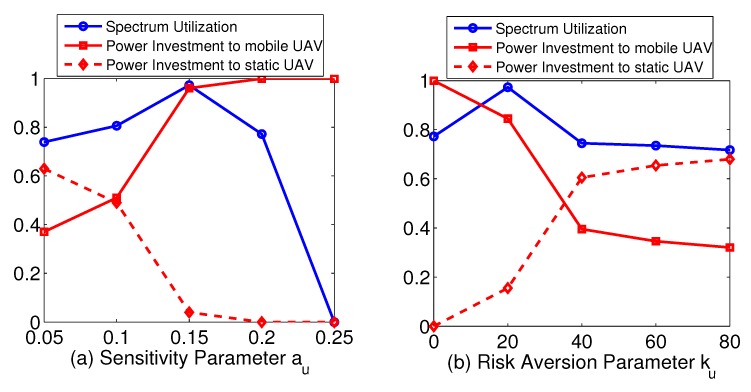
Average spectrum utilization and power investment for varying: (**a**) sensitivity (i.e., parameter $a_{u}$); and (**b**) risk aversion (i.e., parameter $k_{u}$).

We next studied how system utilization is impacted, when a subset of users modify their behavior towards investing in the mobile UAV (which is assumed to have higher spectrum availability), in an attempt to further improve their benefits (i.e., heterogeneous population). Particularly, Figure 3a presents system’s overall spectrum utilization and the respective power investment $x_{u}$ for communicating with the mobile UAV, for a user group that modifies its perceptions towards risk through its prospect-theoretic parameters: (a) sensitivity parameter $a_{u}$; and (b) the risk aversion parameter $k_{u}$. Based on the results, when parameter $a_{u}$ increases, initially this leads to higher levels of investment to the mobile UAV spectrum, while the investment to the static UAV is correspondingly reduced. However, there is a certain level ($a_{u}=0.15$ in our case) where utilization reaches a peak (almost 100%), while subsequently further increasing the sensitivity value makes the users become very aggressive against the mobile UAV spectrum due to its higher expected returns and fully invest in it. As a result, spectrum utilization decreases rapidly as the mobile UAV spectrum collapses due to over-exploitation. At this point, users have fully invested their power to the mobile UAV, as observed by the trend of the corresponding curve presenting the power investment parameter (i.e., $x_{u}=1$), and therefore they do not receive any return from the spectrum of the static UAV either.

Similarly, with reference to the risk aversion parameter, lower values of $k_{u}$ indicate that users are more risk seeking, hence investing more heavily to the mobile UAV spectrum. Consequently, as observed from the results in Figure 3b, when $k_{u}=0$, users do not invest in the static UAV at all, while by rising risk aversion parameter users become more conservative and reduce their investment to the mobile UAV, thus unlocking the additional spectrum of the static UAV, with the utilization eventually reaching 100% at certain point of parameter $k_{u}$. However, for even higher values of $k_{u}$, users keep investing less to the mobile UAV (i.e., CPR) due to their more conservative approach against its probability of failure, and therefore the overall spectrum utilization decreases again.

**Figure 4:**
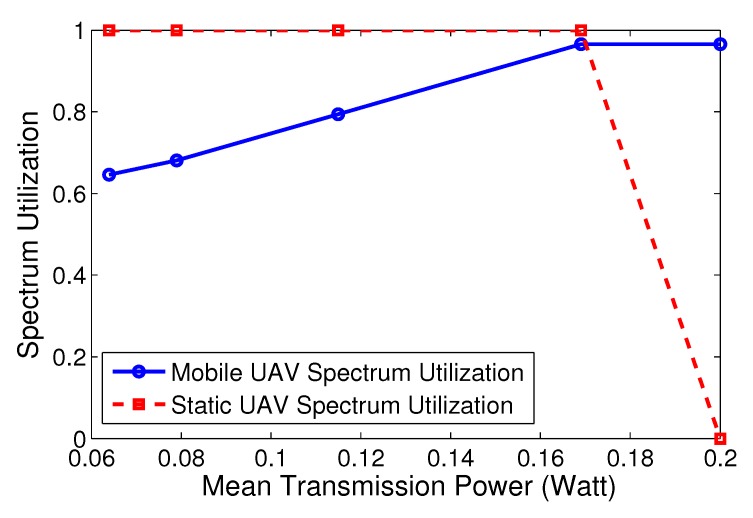
Average mobile and static spectrum utilization per mean transmission power investment.

Additionally, in Figure 4, we demonstrate the achieved spectrum utilization—separately for the mobile UAV and static UAV—as a function of increasing values of transmission power investment to the mobile UAV ($x_{u}$), obtained through properly altering in a combined manner both user prospect-theoretic parameters, that is sensitivity parameter $a_{u}$, and risk aversion parameter $k_{u}$. The combinations of $\left(a_{u},k_{u}\right)$ are (0.05,40),(0.10,40),(0.10,20),(0.15,20),(0.15,10), where the users’ behavior becomes more risk-seeking in the latter choices. For low values of $x_{u}$, users do not invest intensively to the mobile UAV, with the respective spectrum utilization (blue curve) remaining slightly above 60%, while for increasing levels of power, utilization gradually rises until the point that transmission power reaches its upper bound (e.g., 0.2 Watts as assumed here), where utilization to the mobile UAV has reached almost 96.5%. Please note that here we have considered a scenario and respective parameters where the CPR does not collapse. On the contrary, utilization for the static UAV (red curve) follows an opposite trend. For low transmission power levels to the mobile UAV, users invest more in the static UAV, and therefore its utilization remains close to 100%, as an outcome of the safe nature of this resource, as explained before in the paper. In the extreme case where users only transmit via the mobile UAV, users do not opt to communicate with the static UAV, and therefore the respective spectrum utilization eventually drops to zero.

#### 6.1.3. UAV Mobility and Utilization

**Figure 5:**
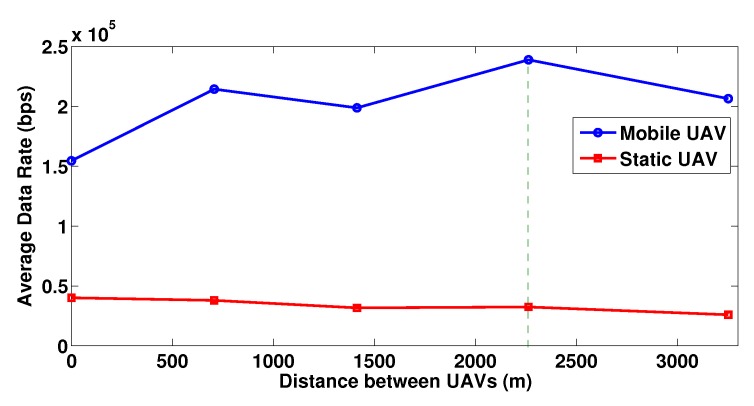
Average user data rate for mobile and static UAV for increasing distance between the UAVs.

In Figure 5, we present the achievable average user data rates for both the mobile UAV and the static UAV, for different snapshots of the system with changing positions of the mobile UAV. The latter is reflected by moving the mobile UAV such that the distance between the static and mobile UAV is increasing (the horizontal axis of this figure reflects exactly this distance). It is expected that the relative position of the mobile UAV against the users in the ground impacts their channel gain conditions. Consequently, this in combination with the application of the NOMA SIC technique, will impact and influence the user decision in their power investment. It is evident that indeed different positions of the mobile UAV result in diverse obtained data rates for the users and the system as a whole, while the average data rate of the static UAV remains practically stable, or is slightly reduced in some cases due to the potential higher investment of the users to the mobile UAV. Specifically, when the distance between the two UAVs reaches approximately 2.3 km, then the majority of the users takes advantage of the most favorable channel gain conditions and they obtain the highest data rates, which are 33.53% higher than the base case (zero distance between the two UAVs).

### 6.2. Comparative Evaluation

In this subsection, the advantages and superiority of the DYNAMISM algorithm compared to other approaches with regards to UAV selection and spectrum utilization is demonstrated and analyzed. In particular, the proposed framework is compared against: (a) an approach where the users follow a fixed UAV allocation under Expected Utility Theory (EUT), which is referred to as EUT-fixed allocation approach; and (b) an approach where users can dynamically connect to the best UAV option (mobile or static) based on their optimal channel gain condition at that specific timeslot. It should be noted that, under the examined comparative scenario of the EUT-fixed allocation approach, each user aims at maximizing its utility function, as expressed by its energy efficiency in Equation 3, where the available bandwidth is y·W for the static UAV and (1−y)·W for the mobile UAV. In the EUT-fixed allocation approach, each user can communicate only with one UAV, i.e., the static or the mobile UAV. The user initially selects the UAV that it will communicate with based on the criterion of the best channel conditions. Then, each user keeps the same communication choice, i.e., the UAV initially selected.

**Figure 6:**
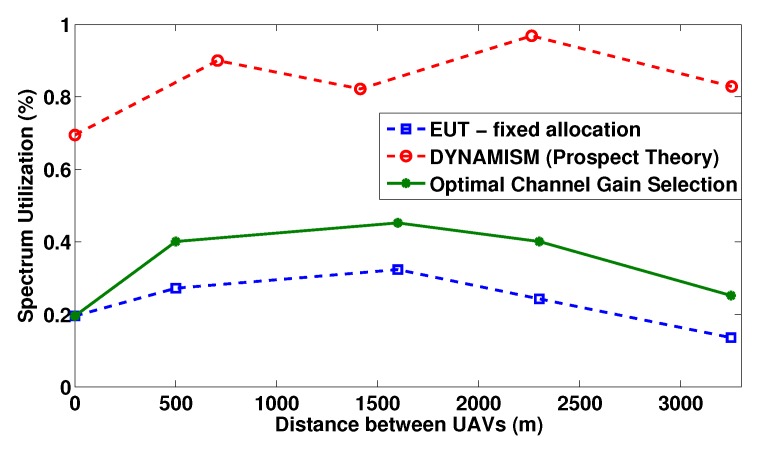
Average total spectrum utilization for increasing distance between the UAVs under different resource management approaches.

Specifically, in Figure 6, we present the average spectrum utilization (from both UAVs) for various positions of the mobile UAV, reflected by the increasing distance between the static and mobile UAV (horizontal axis) for the various approaches considered. Firstly, we notice that the EUT-fixed allocation (blue curve) delivers the worst utilization, since users do not have the capability to dynamically switch between the available bands, and they are forced to stay with the UAV they have been originally assigned to. The approach under which users can dynamically connect to a UAV based on their superior channel gain conditions (green curve), gives an additional degree of freedom to users in order to improve their transmission compared to a fixed allocation and therefore presents a slightly improved utilization compared to the EUT-fixed allocation. Specifically, under the optimal channel gain selection scenario, while the mobile UAV is moving away from the constant position of the static UAV, each user checks the communication channel gain conditions with each UAV and selects to connect with the one that has the best communication channel gain conditions. However, it is noted that this scenario, results in worse spectrum utilization compared to our proposed framework, due to the fact that in the latter the users can jointly exploit their communication with both the UAVs, while in the optimal channel gain selection scenario the user will exploit only the available bandwidth of the UAV that it selected to communicate with, while letting idle and unused the bandwidth of the non-selected UAV. In addition, the optimal channel gain selection scenario leads to better spectrum utilization results compared to the EUT-fixed allocation approach, as in the latter the user keeps its initial UAV selection without dynamically exploiting the channel gain conditions. Lastly, we notice that the DYNAMISM algorithm following the principles of Prospect Theory (red curve), outperforms all other cases, since the users enjoy multiple degrees of freedom with regards to their transmission and are capable of: (a) dynamically splitting their transmission power between both UAVs; (b) investing intelligently in the higher spectrum capacity mobile UAV in a more aggressive manner, while still routing some traffic to the static UAV; and (c) dynamically managing the spectrum utilization of the system in a decentralized and distributed manner, since they are able to modify their behavior based on the risk of the spectrum failure.

The overall performance gains are summarized in Table 2. The proposed prospect theory based approach (DYNAMISM) manages to increase the average user data rate by almost 260% compared to a fixed allocation under EUT-fixed allocation. In addition, note that, although the approach based on the channel gain selection also delivers some improvements (approximately 45%) in the attained user data rates compared to the EUT-fixed allocation, its performance remains significantly lower than the DYNAMISM algorithm.

## 7. Conclusions

In this paper, a user-centric risk-aware resource management and dynamic spectrum management framework is proposed in UAV-assisted PSNs. The PSN is supported by a static UAV and a mobile UAV, where greater portion of the available spectrum is allocated to the latter by the Emergency Control Center due to the potentially better communication channel conditions with the users, as it can dynamically re-position itself and fly closer to the users. The mobile UAV spectrum’s exploitation, while promising higher satisfaction, introduces uncertainty to users’ power investment decisions, as the potentially increased levels of interference if over-exploited can make it fail to serve the users. Furthermore, the resource-constrained environment and the partial information availability introduce risk to users’ decisions regarding their transmission power investment to the static and mobile UAV-based communication.

In this challenging and dynamic environment, in our paper, the users’ risk-aware behavior has been captured following the principles of Prospect Theory and users’ risk-aware prospect-theoretic utility functions have been devised. Respecting the need for developing distributed solutions, a non-cooperative game among the users has been formulated, where each user aims at maximizing its prospect-theoretic utility function by autonomously deciding its power investment to the static and mobile UAV-based communication. The existence and uniqueness of the game’s Pure Nash Equilibrium (PNE) is proven, and convergence to it has been demonstrated. An iterative distributed and low-complexity algorithm is introduced to determine the unique PNE. A detailed series of numerical results, obtained via modeling and simulation, is presented to show the operation of the risk-aware prospect-theoretic resource management framework in terms of achievable data rate and spectrum utilization, under different user behavioral characteristics and scenarios. Moreover, a comparative analysis of the proposed approach against other methods that realize UAV selection and spectrum sharing, has been performed and demonstrated its superiority.

It should be noted that the problem introduced in this paper considers one static and one mobile UAV. Part of our current and future work is to extend this problem by considering multiple static and multiple mobile UAVs. In the latter problem, the users’ optimal uplink transmission power levels at each UAV (to both the static and mobile UAVs) should be determined. This problem is a non-trivial multi-variable non-convex optimization problem that can be addressed as a multi-variable non-cooperative game among the users. Part of this problem is also the positioning of the UAVs within the disaster area, which makes the problem even more complicated. Furthermore, part of our future work is to further exploit the principles of Prospect Theory in studying the problem of distributed denial of service attacks in UAV-assisted PSNs. Finally, the problems of UAV optimal placing while simultaneously determining the number of required UAVs to optimally serve the users, considering the users’ risk-aware behavior, are part of our current and future research goals.

## Figures and Tables

**Table 1:** Simulation Parameter Values.

Parameter	Description	Value
*y*	Portion of the overall available spectrum allocated to the static UAV	0.2 (20%)
*ℜ*	Radius of the PSN	3.5 km
|U|	Number of users	20
*W*	Network’s spectrum	4 MHz
$P_{u}^{Max}$	User’s maximum transmission power	0.2 Watts

**Table 2:** Average user data rate and percent increase in various UAV selection scenarios/approaches.

Scenario/Approach	Average Data Rate per User (bps)	Percent Increase in Average Data Rate
EUT-fixed allocation	$6.55\times 10^{4}$	-
Channel Gain Selection	$9.53\times 10^{4}$	45.50%
DYNAMISM (Prospect Theory)	$23.60\times 10^{4}$	260.22%

## Abbreviations

The following abbreviations are used in this manuscript:

DYNAMISM	DYNAMIc Spectrum Management
UAV	Unmanned Aerial Vehicles
LoS	Line-of-sight
PSN	Public Safety Network
TDMA	Time-Division Multiple Access
TETRA	Terrestrial Trunked Radio
NOMA	Non-Orthogonal Multiple Access
MBS	Macro Base Station
QoS	Quality of Service
ECC	Emergency Control Center
CPR	Common Pool of Resources
PNE	Pure Nash Equilibrium
SLA	Stochastic Learning Automata
SINR	Signal-to-Interference-plus-Noise-Ratio
SIC	Successive Interference Cancellation
RoR	Rate of Return
BR	Best Response
EUT	Expected Utility Theory
